# Excretory/secretory products of anisakid nematodes: biological and pathological roles

**DOI:** 10.1186/s13028-017-0310-3

**Published:** 2017-06-23

**Authors:** Foojan Mehrdana, Kurt Buchmann

**Affiliations:** Laboratory of Aquatic Pathobiology, Department of Veterinary and Animal Sciences, Faculty of Health and Medical Sciences, University of Copenhagen, 1870 Frederiksberg C, Denmark

**Keywords:** Allergy, Anisakidosis, Anisakids, Excretory/secretory products

## Abstract

Parasites from the family Anisakidae are widely distributed in marine fish populations worldwide and mainly nematodes of the three genera *Anisakis, Pseudoterranova* and *Contracaecum* have attracted attention due to their pathogenicity in humans. Their life cycles include invertebrates and fish as intermediate or transport hosts and mammals or birds as final hosts. Human consumption of raw or underprocessed seafood containing third stage larvae of anisakid parasites may elicit a gastrointestinal disease (anisakidosis) and allergic responses. Excretory and secretory (ES) compounds produced by the parasites are assumed to be key players in clinical manifestation of the disease in humans, but the molecules are likely to play a general biological role in invertebrates and lower vertebrates as well. ES products have several functions during infection, e.g. penetration of host tissues and evasion of host immune responses, but are at the same time known to elicit immune responses (including antibody production) both in fish and mammals. ES proteins from anisakid nematodes, in particular *Anisakis simplex*, are currently applied for diagnostic purposes but recent evidence suggests that they also may have a therapeutic potential in immune-related diseases.

## Background

Anisakid nematode larvae of the genera *Anisakis, Pseudoterranova,* and *Contracaecum* (family: Anisakidae; superfamily: Ascaridoidea; order: Ascaridida) are common parasites in a variety of marine fish species worldwide (Table 1). Different species of these parasites have been recognized, while some of them include sibling species within a particular morphospecies, e.g. *Contracaecum osculatum* complex [A, B, C, D, and E] [1], *Anisakis simplex* s.l. [*A. simplex* sensu stricto (s.s.), *A. berlandi* (formerly termed *A. simplex* sp. C) and *A. pegreffii*] [2, 3], and *Pseudoterranova decipiens* complex [*P. decipiens* (sensu stricto), *P. krabbei*, *P. bulbosa* (previously termed *P. decipiens* C) and *P. azarasi* (formerly termed *P. decipiens* D)] [4, 5]. Infection with these parasites is considered a threat to public health due to their zoonotic potential, and the presence of larvae in fish products reduces their commercial value. Free or encapsulated larvae are present within the body cavity, in the visceral organs or in the musculature of the fish host [6] whereby larvae may accidentally be ingested by consumers. The term anisakidosis refers to the disease in humans caused by any member of the family Anisakidae, whereas anisakiasis (or anisakiosis) is specifically caused by members of the genus *Anisakis*, pseudoterranoviasis (or pseudoterranovosis) by the genus *Pseudoterranova* [7, 8] and contracaeciasis (or contracaecosis) is caused by members of the genus *Contracaecum* [9]. Recent studies have revealed that a series of allergens in *Anisakis* play a major role in the progression and clinical picture of the disease. These allergens are a part of a rich series of excretory and secretory (ES) worm products, which may play profound biological roles in the life cycle of these helminths. Research on anisakid ES products has so far mainly focused on *Anisakis* spp., in particular *A. simplex*, owing to its frequent occurrence and cause of anisakiasis. In the present work, we review the biological and pathological role of anisakid ES products with a main focus on the compounds released from the genus *Anisakis*.

**Table 1:** Occurrence of anisakids in fish and humans worldwide

Location	Recorded parasites			Disease form	Ab(s)	Allergen(s) detected	References
	*Anisakis simplex* s.l.	*Pseudoterranova decipiens*	*Contracaecum* sp.				
Spain	+/++	++	+	GA, IA, GAL, AL	mAb UA3 mAb mouse anti-human IgE Rabbit anti-human IgE Goat anti-human IgE	Ani s 1, Ani s 3, Ani s 4, Ani s 5, Ani s 7, Ani s 8, Ani s 9, Ani s 10, Ani s troponin	[54, 62, 64, 65, 68, 69, 75, 76, 77, 85, 86, 94, 115, 120, 121, 148, 149*, 150, 151, 152*, 153*, 154]
Italy	++	++	–	GA, IA, GAL, AL	Goat anti-human IgE mAb mouse anti-human IgE	Ani s 1, Ani s 4, Ani s 5, Ani s 9, Ani s 10	[23, 24, 105, 116, 155, 156]
Norway	+/++	+	+	AL	mAb mouse anti-human IgE	Ani s 1, Ani s 7	[122, 157*, 158*, 159*]
Denmark	+/++	+	+	IA	–	–	[146*, 160, 161*, 162*]
Sweden	–	+	–		–	–	[163*]
Iceland	–	++	–	GA	–	–	[31]
Germany	++	–	++	GA, GIA	–	–	[27, 164]
Netherlands	++	–	–	GA	–	–	[165]
Poland	+	+	+		–	–	[147*]
Croatia	++	–	–	AL	NA	Ani s 1, Ani s 7	[166]
Portugal	+	–	–		–	–	[167*]
France	++	–	–	GIA	–	–	[168]
Japan	+/++	++	+/++	GA, AL	Goat anti-human IgE	Ani s 8, Ani s 9, Ani s 11, Ani s 12	[29, 57, 66, 83, 169, 170*, 171*]
Korea	++	++	–	GA, GIA, AL, GAL	NA	NA	[21, 25, 89, 172, 173]
Taiwan	++	–	–	GA	–	–	[129]
Canada	++	+	–	IA	–	–	[174*, 175]
USA	++	++	+	GA, IA	–	–	[176, 177, 178*]
Brazil	+	+	+		HRP-mouse anti-human IgE	NA	[179, 180*, 181*]
Chile	+	+/++	–	GA	–	–	[26, 182*, 183]
Argentina	–	–	+		–	–	[184*]
Australia	+	–	+/++	GIA	–	–	[28, 185*]
Egypt	+	+	+		–	–	[6*]
South Africa	++	–	–	AL	NA	NA	[52]

+ infection recorded in fish, ++ infection recorded in humans, *Asterisk sign* (*) studies reporting on occurrence of the parasites in fish populations, *GA* gastric anisakidosis, *IA* intestinal anisakidosis, *GIA* gastrointestinal anisakidosis, *AL* allergy, *GAL* gastro-allergic anisakiasis, *NA* not available, *Ab(s)* antibodies used for *Anisakis*-specific IgE detection in patients sera

## Search strategy

A literature search was conducted in PubMed (http://www.ncbi.nlm.nih.gov/pubmed) and ScienceDirect (http://www.sciencedirect.com) using the terms “excretory and secretory products” AND “allergy” OR “anisakidosis” combined with anisakid parasites names “*Anisakis*” OR “*Pseudoterranova*” OR “*Contracaecum*”. The title and abstract of resulted hits were evaluated and the most relevant articles were assessed in detail. Our own archives were also used as an additional source of information. The papers included in this systematic review have been published between 1960 and 2016.

## General biology of anisakids

The life cycles of anisakid nematodes comprise adult worms in marine mammals, e.g. seals, sea lions, dolphins, whales [7, 10, 11] and/or piscivorous birds [12–14] and hatched larvae which are free-living until they are ingested by an invertebrate host (e.g. a crustacean) whereafter they are transferred to a teleost transport host by predation. Humans act only as accidental hosts for anisakids. They obtain infection through consumption of raw or underprocessed seafood, but the nematodes do not reach the adult stage in humans whereby human hosts cannot transmit the infection further by releasing parasite eggs with feces. In contrast, marine mammalian hosts (pinnipeds and cetaceans) allow maturation of the anisakid worms in their gastrointestinal tract. Following copulation between adult male and female worms, parasite eggs are released by the adult female worm and leave the host with faeces to the marine environment where they develop and subsequently hatch [15]. The released free third stage larvae (L3) become ingested by the first invertebrate hosts (including crustaceans, cephalopods and polychaetes) in which they reach extra-intestinal sites such as the hemocoel, a process which must involve enzymatic activity. Following ingestion by the fish, the worm larvae penetrate the fish gut and reach internal organs such as body cavity, viscera or musculature. The fish host range depends to some extent on the anisakid species [2, 13, 16] but their geographical distribution is also limited by the availability of the intermediate and final hosts [17]. Therefore, the presence of the parasite in a host implies the co-presence of all the required host species for completing the parasitic life cycle at the same time in the same area and indicates that ES genes encoding products needed for all steps in the life cycle are present in that particular strain of the parasite [18].

## Human infections

Humans are accidental hosts of anisakid parasites, and acquire L3 through consumption of raw or inadequately processed seafood. Ingestion may cause anisakidosis, which is manifested by distinct gastrointestinal symptoms, e.g. vomiting, diarrhoea, and epigastric pain [19, 20]. *Anisakis simplex* s.s. (Rudolphi, 1809) is the most frequently reported causative agent for anisakiasis [8] but recently *Anisakis pegreffii* was reported to cause anisakiasis in the Republic of Korea [21], Croatia [22], and Italy [23, 24]. Infections caused by *P. decipiens* (Krabbe, 1878) [25, 26] and *C. osculatum* (Rudolphi, 1802) [27–29] have been reported at a lower frequency (Table 1). Infections with *Pseudoterranova* may in certain cases cause asymptomatic infections and come to medical attention only when worms are recovered following vomiting, coughing or defecating [30, 31]. The few cases of contracaeciasis reported severe abdominal pain associated with the infection [27, 28].

## Production of ES compounds

During all stages of the life cycle, nematodes produce and release a series of excretory and secretory molecules (ES compounds) which may be key players in parasite-host interactions including host-specificity. However, this does not necessarily mean that the composition of compounds or the individual molecules are identical at all stages [32]. It may be suggested that production of ES compounds in the third stage larvae varies (quantitatively and qualitatively) depending on the type of host (crustaceans, fish and mammals) due to the different structural and physiological conditions in these host groups. The habitat of poikilothermic organisms, such as crustaceans and fish, may reach near zero degree in certain marine areas whereas marine mammals are homoiothermic animals with body temperatures near 40 °C, which challenges the temperature optima of enzymatic systems differently. Thus, the temperature-dependent production of ES compounds in *Anisakis* was shown by Bahlool et al. [33]. In addition, the chemical interactions (such as receptor-ligand binding) between host and parasite must differ due to conformational changes of proteins at different temperatures. A number of genes encoding central immune factors have been partly conserved throughout evolution from invertebrates via fish to mammals, but the variation is high [34, 35] and thereby it should be expected that host evasion mechanisms in different animal groups differ. It has also been suggested that differences among life cycles of different parasite species and even sibling species [11, 36] may be attributed to the relative abundance and function of these bioactive molecules influencing host specificity [37].

## Biochemical composition of ES products

The ES molecules can be released from parasite organs including glands, oesophagus, ventricle, intestine and outer surfaces. In the final host, adult male and female worms mate and it is believed that during this phase chemical communication occurs between sexes which may add sex pheromones to the list of possible ES products. At all stages various enzyme activities have been associated with the released materials. Enzymes serving a basic metabolic role in the parasite, acid and alkaline phosphatases are found [33] and together with enzymes connected to infectivity, immune evasion and pathogenicity (proteases, nucleotidases, esterases, glycases, dismutases) they may serve roles at all life cycle stages. However, no studies have as yet been presented showing the action of ES products in invertebrate hosts and it cannot be excluded that different isotypes are expressed to different degrees in intermediate and final hosts. It is known that hydrolytic enzymes enable the worm to penetrate and migrate in fish tissues [33] and several other functions have also been suggested for secreted proteins from nematodes. For example, some anticoagulant activities are recorded from larval *A. simplex* ES products causing prolongation of partial thromboplastin time (PTT) which may have a key role in human anisakiasis regarding larval penetration into the gastrointestinal mucosa [38]. Moreover, a number of ES compounds from *A. simplex* larvae ranging from 66 to 95 kDa may have a cytostatic inhibitory effect on lymphocyte blastogenesis [39]. Acetylcholinesterase (AChE) released by some gastrointestinal nematodes may play an important role in altering permeability of host intestinal cells to secure parasite feeding and therefore survival. This enzyme may also adversely affect coagulation and glycogenesis in the host [40]. Podolska and Nadolna [41] speculated that increased secretion of AChE from *A. simplex* larvae in herring should be considered an adaptive response to neurotoxic compounds released by the host. In general, nematode secretions have immunomodulatory effects interfering with host immune responses. AChE, glutathione-*S*-transferase (GST), and superoxide dismutase (SOD) secreted by the hookworm *Necator americanus* are known to suppress host inflammatory responses [42]. This is in line with secreted AChE from the filarial nematode *Wuchereria bancrofti* where the suppressive effect is due to degradation of acetylcholine, a neurotransmitter, which is responsible for releasing lysosomal enzymes and phagocytosis in the host [43]. AChE produced by the ruminant nematodes *Ostertagia* and *Haemonchus* has been assumed to affect host responses by controlling gastric acid secretion [40]. GST has been identified in secretions from the swimbladder nematode *Anguillicoloides crassus* in European eels and its function was suggested to quench reactive oxygen radicals released as part of the host innate responses toward the infection [44]. Proteolytic enzymes produced by *A. simplex* larvae are likely to target central proteins in the teleost immune system, e.g. antibodies and complement factors, and thereby enhance the parasite survival in the fish [33].

Future proteomic studies are likely to extend the list of annotated molecules in the ES molecule mixture of anisakids but it may be worthwhile to search molecules already described from a range of parasites (see the review [37]). Thus, apart from a range of enzymes and antioxidants, functional effector molecules including protease inhibitors, lectins, heat shock proteins, mucins and cytokine regulators may be detected.

## Immunogenicity of ES products

Many of the *A. simplex* ES molecules are highly immunogenic and can provoke antibody production both in fish and mammals. Serum obtained from infected saithe (*Pollachius virens*) were found to react with larval *A. simplex* molecules in an enzyme linked immunosorbent assay (ELISA) [45], and specific antibodies from European eel (*Anguilla anguilla*) reacting against GST in ES isolated from *A. crassus* were detected by western blotting [44]. ES molecules in other anisakid larvae have not been studied to the same extent, but several proteins from *Contracaecum* species have been isolated and shown to elicit a humoral response in Antarctic teleosts [46]. Seals also produce antibodies with affinity to anisakid antigens. In a study focusing on seal serum antibody reactivity against the adult lungworm *Otostrongylus circumlitus*, it was found that the sera also reacted with whole body extract of other nematodes including *Pseudoterranova* sp. and *Anisakis* sp. [47]. This corresponds to the well-studied antibody production in mammals against nematode antigens, which even has been found associated with protective immunity [48, 49]. The humoral immune reactions against ES products from *A. simplex* in accidentally infected humans have been intensely investigated. Several immunoglobulin classes may be involved, but worm specific IgE has attracted considerable interest because it is associated with disease progression and allergic responses to the parasite.

## Allergenicity of ES products

Symptoms associated with anisakid nematode larvae present in human tissues may—at least in some cases—be due to allergic responses. Allergens in *A. simplex* comprise both somatic antigens (SA) and ES molecules and several have been shown to be resistant to various freeze-, heat- and digestive processes. It is believed, based on empirical data, that allergy towards *A. simplex* must be induced by an active infection by a live worm but then subsequent exposure to allergens including ES products is sufficient to elicit an allergic response [50]. However, ingestion of larvae is not the only possibility to acquire anisakid-related disease. Occupational exposure to the parasitized fish containing anisakid allergens can elicit allergic reactions, e.g. bronchial hyperreactivity and dermatitis [51–53].

### Anisakis allergens

*Anisakis simplex* has so far been described as the only anisakid parasite responsible for allergic reactions in humans. Different groups of allergenic molecules have been isolated from L3 larvae; (1) ES proteins secreted by the parasite, (2) SA of the larval organs, and (3) cuticular proteins [8]. Allergenic proteins (Ani s1 to Ani s12, Ani s 13, Ani s 14, Ani s 24 kDa, Ani s CCOS3, Ani s cytochrome B, Ani s FBPP, Ani s NADHDS4L, Ani s NARaS, Ani s PEPB, and Ani s troponin) have been described in *A. simplex*, of which Ani s 1, Ani s 2, Ani s 7, Ani s 12, Ani s 13, Ani s 14, and an Ani S 11-like protein (Ani s 11.0201) are identified as major allergens [54–60]. Allergens Ani s 7 and Ani s 10–12 are still uncharacterized with unknown functions [54]. A number of putative novel allergens (cyclophilin and two proteins with unknown function) have recently been characterized for the first time from *A. simplex* transcriptomes by comparing predicted amino acid sequences with homologous known allergenic proteins [61]. In general, *A. simplex* ES allergens are known to be more potent which could be a result of their higher affinity to specific IgE compared to the somatic antigens [62].

### Allergen persistence

Despite the fact that anisakid larvae lose their infectivity by adequate food preparation, it should be noted that parasite allergens (SA or ES products) may be resistant to heat, freezing, and pepsin (Ani s 1, Ani s 4, Ani s 5, Ani s 8, Ani s 9, Ani s 10, Ani s 11.0201) as they preserve the antigenicity and may trigger allergic responses in sensitized persons following consumption of well-cooked or canned fish [60, 63–70].

### Allergen cross-reactivity

IgE raised in patients against SA and ES antigens of *A. simplex* may cross-react with homologous antigens of other ascarid nematodes (e.g. *Ascaris suum*, *Ascaris lumbricoides, Toxocara canis*, *Hysterothylacium aduncum*), or arthropods (German cockroach, chironomids) [71–73]. However, somatic proteins are more likely to cross-react, while ES antigens are more specific. For example, Ani s 2 (paramyosin, a somatic antigen) has been shown to have high similarity and, therefore, high degree of cross-reactivity with some dust mites, e.g. *Acarus siro* and *Tyrophagus putrescentiae*. Ani s 3 (tropomyosin), another somatic allergen, is also suggested to have the potential to cross-react with molecules from crustaceans, e.g. *Homarus americanus* (American lobster), and *Metapenaeus ensis* (greasyback shrimp), molluscs, e.g. *Perna viridis* (green mussel), and *Crassostrea gigas* (giant Pacific oyster), and also with the insect American cockroach (*Periplaneta americana*) [74]. The allergen Ani s 1, an ES protein, is generally considered to have no cross-reaction with other allergens, which make it a suitable candidate for diagnosis of hypersensitivity and intestinal anisakiasis [75, 76]. Using this allergen along with Ani s 4 has been shown to achieve a diagnostic sensitivity of 95% by IgE immunoblotting [77]. Further precision of diagnosis may be achieved if combined with detection of Ani s 5, another ES antigen, which also has demonstrated its utility for serodiagnosis of the *Anisakis* larvae sensitization [68].

### Allergens in other anisakids

The allergenic potential of other anisakids, e.g. *P. decipiens*, molecules has not been studied to the same extent as *A. simplex*. A number of somatic antigens in *C. osculatum* larvae have been isolated with the molecular weight of 47, 63, and mainly 91 kDa [46], but a recent study using experimental infection of mice with live *Contracaecum* sp. larvae did not show IgG or IgE antibody responses specific to SA or ES antigens [78]. However, the *Contracaecum* body structure and migratory strategy in the fish host are partly similar to those of *Anisakis* larvae [79] suggesting that further genomic and proteomic analysis of SA and ES molecules of *Contracaecum* L3 should be conducted.

## Pathology and ES products

Pathological changes associated with anisakidosis may result from the direct tissue invasion by the larva into the gastric or intestinal mucosa, but immunological reactions (cellular and humoral) towards worm constituents are likely to play a major role. It has been suggested that the parasite pathogenicity may vary among closely related species and geographic strains [80–82] which may at least partly explain differential occurrence of disease. In addition, the infection dosage may be expected to influence the host reaction. In many cases of anisakidosis a single larva is responsible for infection. However, a total of 56 *A. simplex* larvae were recovered in a patient in Japan [83], and another human case in Spain was diagnosed infected with more than 200 *A. simplex* larvae accumulated in the gastric mucosa [84].

Clinical symptoms are partly connected to allergic reactions involving IgE-mediated hypersensitivity with resulting acute urticaria, angioedema, and anaphylaxis occasionally accompanied by gastroallergic anisakidosis [8, 85–89]. However, specific anti-*Anisakis* IgE is still detectable in patients over the years after the allergic episodes with a declining trend [90].

Cellular reactions with partial remodeling of tissues involving infiltration with macrophages, eosinophils, mast cells, neutrophils and lymphocytes at the penetration site are known to occur both in fish and pigs [33, 91]. Furthermore, in a recent in vitro study exposure of human fibroblast cell line HS-68 to *A. pegreffii* ES compounds led to elevation in reactive oxygen species (ROS) levels causing oxidative stress and also activation of kinases and subsequent inflammation, cell proliferation, inhibition of apoptosis and DNA damage [92].

In the case of invasive anisakidosis, ulcerations and hemorrhages are found in the intestinal or stomach wall. Even if worm larvae die in the human host, it should be noted that antigens released from the remains of the worm may induce inflammatory responses eliciting symptoms which cannot be differentiated from other disorders, e.g. cholecystitis, neoplasia, gastritis, peritonitis [93], appendicitis [94], eosinophilic gastroenteritis, and Crohn’s disease [95].

## Diagnosis and ES products

Diagnosis of anisakidosis initially relies on a detailed history of recent seafood consumption and may be confirmed by direct visualization and examination of the larvae. Removal of the worm by endoscopy/colonoscopy [96] or surgery [97] allows concurrent diagnosis and treatment of gastric/intestinal form of the disease, but non-invasive methods such as sonography and X-ray have also been proven as valuable diagnostic tools [98–100]. Haematological evaluations may show leukocytosis, e.g. mild to moderate eosinophilia, and mast-cell degranulation [93, 101, 102]. Diagnosis of anisakiasis can be conducted with serologic tests which are partly based on reactions towards ES products of the worm. ELISA, IgE immunoblotting and ImmunoCAP can detect *Anisakis*-specific IgE reactivity to a complete extract of *Anisakis* L3 larvae which supports diagnosis of intestinal and allergic diseases [75, 103–105]. However, interpretation of results may not be clear-cut due to cross-reactivity of the *A. simplex* antigens with other antigens such as products from *Ascaris* spp., *T. canis*, insects (cockroaches) or crustaceans (shrimps) and care should be taken to omit false-positive serology results [106–108]. Since it has been shown that detection of specific IgG4 raised in the infected human host against *A. simplex* is likely to be more specific than specific IgE in diagnosis of gastro-allergic anisakiasis [88, 109], detection of this Ig subclass is relevant to include in serological tests. Flow cytometry has also been applied as a tool for diagnosing allergy to *Anisakis* products activating basophils [110]. Skin prick tests (SPTs), inserting *Anisakis* products into the skin of the patient, may assist diagnosis of the allergic form of the disease mediated by cellular immune responses, but the test has a low specificity and high rate of false positives due to cross reactivity with other allergens from seafood and mites [111], and from *A. lumbricoides* [112, 113]. This frames the necessity of improving diagnostic kits based on specific *Anisakis* antigens, e.g. purified natural or recombinant allergens [114–116] and has accelerated immunoscreening of protein-expressing cDNA libraries [117], phage display system [118], and mass spectrometry-based proteomics [54] to identify novel allergen candidates.

It has been shown that the application of recombinant allergens of *A. simplex*, expressed in *Escherichia coli* or *Pichia pastoris*, can improve diagnostic assays by increasing specificity and avoid misdiagnosis caused by cross-reactions [115]. Measuring IgE reactivity to recombinant Ani s 1 (rAni s 1) and Ani s 7 (rAni s 7) allergens has been suggested as the most efficient serodiagnostic means for anisakiasis, when combining sensitivity and specificity. However, Ani s 1 is considered the major allergen in gastro-allergic anisakiasis, while Ani s 7 can be recognized independently of the amount of specific IgE production, i.e. in the case of chronic urticaria with lower serum specific IgE values [119, 120]. Furthermore, an internal fragment of the rAni s 7 (435Met-713Arg), known as t-Ani s 7, is shown to have the potential to improve serodiagnostic specificity [121]. In a recent survey of two groups of subjects in Norway, including recruited blood donors (BDO) and patients with total IgE levels ≥1000 kU/l (IGE+), the prevalence of anti-*Anisakis* IgE antibodies was 0.4 and 16.2% in the BDO and IGE+ groups, respectively. However, further analyses of *Anisakis* positive sera by ELISA against recombinant allergens rAni s 1 and rAni s 7 showed a seroprevalence of 0.0 and 0.2%, respectively, and it cannot be excluded that false-positivity occurs due to cross-reactivity to other allergens such as shrimp and house dust mite [122]. Gamboa et al. [123] also emphasized the value of rAni s 1 for diagnosing allergy to *Anisakis* both in vivo (SPT) and in vitro [specific IgE and basophil activation test (BAT)]. Both natural and recombinant Ani s 10 have also shown positive reactivity with 39% of *Anisakis*-allergic patients’ sera [69]. Besides high specificity, there are other advantages using recombinant allergens. For example, the yield of purified recombinant *Anisakis* proteins from bacterial cultures is higher compared to the yield of the natural protein from *Anisakis* larvae, while they show equivalent immunochemical properties [124, 125]. Asturias et al. [126] reported a high yield of 6.6 mg/L culture of a purified recombinant tropomyosin from *A. simplex* (*As*-TPM), whereas the final yield of the purified natural *As*-TPM was only 0.36 mg/g of *Anisakis* larvae, which advocates for inclusion of recombinant allergens in allergy diagnostic tests.

## Treatment and ES products

There is no standard medication available to treat anisakiasis. However, benzimidazoles such as the anthelmintic albendazole (400–800 mg daily for 6–21 days) have been suggested as a possible therapy [127–129]. It has also been shown that administration of corticosteroids like 6-methylprednisolone (1 mg/kg/24 h for 5 days) may be a useful option to treat the acute intestinal anisakiasis as an alternative to surgical resection [130]. Moreover, prednisolone (5 mg/day for 10 days) and olopatadine hydrochloride (10 mg/day for 6 weeks) have demonstrated promising results to resolve intestinal anisakiasis symptoms [100].

In addition, novel treatment options are likely to follow. Thus, in vitro studies on larvicidal activities of natural terpenes, e.g. geraniol, citronella essential oil, and tea tree essential oil [131, 132], *Matricaria chamomilla* essential oil (including α-bisabolol) and in vivo work on administration of the aldehydic monoterpene citral and the alcoholic citronellol suggested that these compounds may be effective against infections caused by *A. simplex* and/or *Contracaecum* sp. [133–136]. Medical treatment leading to killing worm larvae in tissues may result in significant release of worm antigens (SA and/or ES products) which could exacerbate disease symptoms and it may be necessary to combine treatment with immune-moderating drugs such as corticosteroids.

## Therapeutic potential of anisakid molecules

Ascarid nematode larvae carry genes encoding various immunoregulatory products which ensure the survival of the parasite in the host immune environment [137, 138] and ES products of anisakids are expected to have similar properties. In a mouse experimental model of asthma, induced by an *A. suum* allergen (APAS-3), it was shown that an ES protein, PAS-1, could reduce Th2 responses, inhibit cellular migration, suppress cytokine expression (IL-4, IL-5), and reduce chemokine production in bronchoalveolar lavage (BAL) fluid [139]. Similarly, PAS-1 has in a mouse model been shown to have an inhibitory effect (probably mediated by IL-10 and TGF-β secretion) on *E. coli* LPS (lipopolysaccharide)-induced inflammation via suppression of TNF-α, IL-1β and IL-6 [140, 141]. Lung allergic inflammation in mice induced by ovalbumin (OVA) was inhibited by PAS-1 immunization mediated by stimulation of IL-10 and IFN-γ production and subsequent suppression of cytokine and antibody reactions [142, 143]. An anaphylactic immune response to peanut in a mouse model has also been inhibited partially by *A. simplex* or *A. lumbricoides* somatic extracts through reduction of specific IgG1 and subsequently inhibition of anaphylactic symptoms score [144]. It was also shown by Bahlool et al. [33] that *Anisakis* ES compounds decreased expression of genes encoding inflammatory cytokines. In addition, a recent study has demonstrated immunoregulatory effects of *A. simplex* ES antigens in a colitis zebrafish model [145]. These findings suggest that by appropriate biochemical techniques the immunoregulatory potential of anisakid ES molecules may be further characterized and exploited for prevention and/or treatment of inflammatory diseases.

## Conclusion and perspectives

Increasing population of anisakid final hosts (marine mammals) and thereby their endoparasitic anisakid nematodes may lead to elevated infection levels in fish [146, 147]. This may together with the increasing trend of raw or undercooked seafood consumption explain increasing occurrence of anisakidosis and infection-induced allergies. ES products released by the anisakid nematodes have been shown to play a central role not only in the general biology of the parasite but also in human disease. Some ES products elicit allergic responses in humans but as in other helminths, other ES products may modify host immunity and suppress immune responses which open alternative usage of anisakid parasite products as therapeutics. In this review, we have focused on *A. simplex* allergens and the associated allergy, since our current knowledge is mainly limited to this species. The immunomodulatory activities of other relevant anisakids, particularly *P. decipiens* and *C. osculatum*, are still inadequately described and further investigations using in vitro and in vivo techniques are necessary to identify the allergenic or immunosuppressive properties of anisakid-originated components and elucidate the mechanisms involved in immunoregulations.

## Abbreviations

AChE: acetylcholinesterase
As-TPM: *Anisakis simplex* tropomyosin
BAL: bronchoalveolar lavage
BAT: basophil activation test
BDO: blood donors
ELISA: enzyme linked immunosorbent assay
ES: excretory and secretory
GST: glutathione-*S*-transferase
L3: third stage larvae
LPS: lipopolysaccharide
OVA: ovalbumin
PTT: partial thromboplastin time
rAni s 1: recombinant Ani s 1
ROS: reactive oxygen species
SA: somatic antigens
SOD: superoxide dismutase
SPT: skin prick test
